# The Moderating Role of Regulatory Institutional Environment in the Relationship Between Emotional Job Demands and Employee Absenteeism Likelihood of Healthcare Workers. Evidence From the Low-Income Country Context

**DOI:** 10.3389/fpsyg.2020.01052

**Published:** 2020-05-26

**Authors:** Benson Munyenyembe, Ying-Yu Chen, Wen-Chiung Chou

**Affiliations:** ^1^Department of Business Administration, College of Management, National Dong Hwa University, Hualien, Taiwan; ^2^Bachelor Program of Management Science and Finance, College of Management, National Dong Hwa University, Hualien, Taiwan; ^3^International Honors Bachelor Program, College of Business, Kainan University, Taoyuan, Taiwan

**Keywords:** absenteeism likelihood, emotional job demands, healthcare workers, low-income country, regulatory institutional environment, work engagement

## Abstract

Previous research has not clearly studied how the effects of emotional job demands on absenteeism likelihood are moderated by the contingent absenteeism-related regulatory institutional environments of low-income countries. In this regard, we surveyed 487 healthcare workers in a low-income country in order to test for the effect of emotional job demands on healthcare workers’ absenteeism likelihood. We also explored the mediating role of work engagement and the contingent role of context-specific regulatory institutional environments on the link between emotional job demands and absenteeism likelihood. The main findings of this study are as follows: (1) emotional job demands have a direct positive effect on healthcare workers’ absenteeism likelihood, (2) work engagement plays a mediating role on the link between emotional job demands and healthcare workers’ absenteeism likelihood, and (3) the regulatory institutional environment related to absenteeism moderates the negative link between work engagement and absenteeism likelihood. Results in this study demonstrate the crucial role that the context-specific regulatory institutional environment related to absenteeism plays in suppressing the effect of emotional job demands on absenteeism likelihood when considered through the work-engagement pathway. The study’s findings clarify the mechanism through which emotional job demands affect absenteeism likelihood in a low-income country context. The study thus offers a new refined theoretical perspective on how emotional job demands, work engagement, and context-specific regulatory institutional environments interact in ways that predict absenteeism likelihood.

## Introduction

In the low-income country context, the issue of employee absenteeism is of vital significance because it presents one of the major challenges that organizations face. Even with the availability of official rules providing for possible punitive action in the case of repeated absenteeism, actual enforcement of such official rules has been found to be loose in low-income countries, complicating the problem of rampant employee absenteeism (Chaudhury et al., 2006). Low-income countries have also been characterized by “professional human resource” crises due to the limited availability of adequately trained employed personnel – an issue that underscores the importance of the employee-absenteeism problem (Belita et al., 2013). Employee absenteeism in the low-income country context is regarded as one of the biggest human-capital risks to productivity-improvement goals (Ejere, 2010). Also important to note is that over the past decade, voluminous literature on employee absenteeism has sprung up offering a variety of conceptual models and theories, most of which are constructed from the Anglo-American and Euro-Asian contexts, not low-income country contexts. The scant research has created an ideal opportunity for researchers to systematically document the practices of employee absenteeism in the context of the low-income country (Molleda and Moreno, 2008).

In particular, a general consensus that has emerged points out that the regulatory institutional environments in low-income countries are weak and significantly different from the ones present in developed and emerging economies (Khanna et al., 2005; Meyer and Peng, 2005; Wright et al., 2005; Chaudhury et al., 2006; Gelbuda et al., 2008). Given these regulatory institutional-environment differences, how do emotional job demands affect employee-absenteeism likelihood in the low-income country context? And in this context, does the regulatory institutional environment related to absenteeism play any contingent role in the link between emotional job demands and employee-absenteeism likelihood? On the basis of these research questions, we have adopted the job demands-resource (JD-R) model to understand the effect of emotional job demands on employee-absenteeism likelihood in the low-income country context. We have incorporated the regulatory institutional environment into the JD-R model to test for the contingent role that a low-income country’s regulatory institutional environment plays in the link between emotional job demands and employee-absenteeism likelihood.

Employee absenteeism emphasizes the idea that, in normal workplace circumstances, employees can avoid being absent (Avey et al., 2006). This view thus asserts that employee absenteeism represents an optional, or voluntary, behavior unapproved by the organization where the employee chooses not to report for work. As a multidimensional phenomenon, employee absenteeism involves various causes that have been discussed in the literature. Many studies setting out to determine the complex patterns predictive of absenteeism have used predictors of absenteeism relating broadly to personality factors, attitudinal factors, demographic factors, health-related factors, organizational factors, labor-market conditions, and job characteristics (Ćikeš et al., 2018). Other studies have linked various types of job demands to various employee counterproductive work behaviors including employee absenteeism (Grieco, 1987; Robinson and Bennett, 1995; Penney et al., 2011; Appelbaum et al., 2012; Ahmed et al., 2013; Chen et al., 2017). However, no study to our knowledge has incorporated the regulatory institutional environment into the JD-R model in order to clarify (1) how emotional job demands affect employee absenteeism likelihood and (2) how the context-specific regulatory institutional environment moderates the link between emotional job demands and employee absenteeism likelihood.

Linking the JD-R model to the absenteeism of healthcare workers in the context of a low-income country is captivating for two reasons. First, the healthcare-worker density in most low-income countries is well below the World Health Organization (WHO) recommended minimum of 2.5 healthcare workers per 1,000 populations while the burden of disease is high (Kombe et al., 2005; Liu et al., 2017). With such low health care-worker density, the problem of healthcare workers’ absenteeism is thus hugely more significant in low-income countries than in developed countries, hence triggering – in the former countries – not only a loss of man hours and a loss of productivity, but also a loss of patients’ lives (Oche et al., 2018). In recent years, low-income countries have been challenged with the task of being required to nearly triple their number of healthcare workers, adding some 1 million of them to the existing ranks so as to meet domestic demand (Chen et al., 2004; Liu et al., 2017). Second, in low-income countries, the emergence and re-emergence of infectious diseases such as HIV/AIDS, TB, and malaria have increased demand for health services, putting an additional stress on the countries’ already small number of healthcare workers (Richard et al., 2016). With such increased demands for health services coupled with low healthcare-worker density per 1,000 populations, healthcare workers in low-income countries continue to be overwhelmed in a way that makes high job demands a daily operational routine (Dieleman et al., 2009).

Researchers have widely applied management literature’s JD-R model to research on employees’ counterproductive work behavior, one significant example of which is absenteeism (Schaufeli and Bakker, 2004; Xanthopoulou et al., 2009). Some previous research has proved that work engagement, job anxiety, psychological detachment, burn-out, and job stress play intermediary roles in the link between job demands and counterproductive work behaviors (Schaufeli and Bakker, 2004; Xanthopoulou et al., 2009; Chen et al., 2017). However, the role of the regulatory institutional environment has been ignored as a possible contingency factor in the link between emotional job demands and employee-absenteeism likelihood when looked at through the work-engagement pathway. To our knowledge, no previous research integrated regulatory institutional environments into the JD-R model to test if this factor plays a contingent role in the link between emotional job demands and employee-absenteeism likelihood. The scant research on this possible role particularly in the low-income country context is what prompted the current study.

Overall the current research makes three significant contributions to the literature. First, this study enriches the JD-R model as a way to better understand employee-absenteeism likelihood in the low-income country context. Our primary hypothesis suggests that emotional job demands positively relate to employee-absenteeism likelihood. Second, the current research incorporates and clearly demonstrates the moderating role that absenteeism-related regulatory institutional environments play in the effect of emotional job demands on employee-absenteeism likelihood. We achieved this objective by testing the moderating role of regulatory institutional environments on the link between emotional job demands and employee-absenteeism likelihood when this link is considered through the work-engagement pathway. The current research should significantly contribute to the literature regarding the role that context-specific regulatory institutional environments play in employee absenteeism. Finally, by featuring a primary survey in a low-income country, the current research extends the geographical reach of other research on employee absenteeism in the low-income country context from both an emotional-job-demand perspective and a regulatory-institutional-environment perspective. Because the low-income country context is quite different from the contexts of developed and emerging economies, the current research sheds additional light on how emotional job demands and the regulatory institutional environment interact with each other in ways that help predict employee-absenteeism likelihood in the low-income country context. Snejina (2011) stressed the need to conduct contextualized research for the purposes of generating accurately generalizable empirical results. The research community therefore recognizes the need for continued systematic country-by-country documentation of employee practices (Molleda and Moreno, 2008; Xu and Meyer, 2012).

## Theoretical Basis and Hypothesis Development

### Emotional Job Demands and Absenteeism Likelihood

Emotional job demands are those psychological or social aspects of a job that require sustained emotional effort and are therefore associated with certain psychological costs (Demerouti and Bakker, 2011). The effect of emotional job demands on employee absenteeism is better explained by the JD-R model, which has gained prominence in recent years and reflects an effort to accommodate two traditional research traditions: the “stress research tradition” and the “motivation research tradition” (Azharudeen and Anton, 2018). The key argument raised under the JD-R model is that although emotional job demands are not necessarily negative, they may turn into job stressors once employees come into contact with emotional demands that require a high expenditure of emotional effort from which the employee fails to recover adequately (Meijman and Mulder, 1998). Emotional job demands, when present in high amounts, are likely to become a source of emotional exhaustion for employees, hence leading to employee absenteeism. Accordingly, we propose the following hypothesis.

Hypothesis 1: There is a positive link between emotional job demands and absenteeism likelihood.

### The Mediating Role of Work Engagement

Work engagement has been defined as a positive, fulfilling, work-related state of mind that is characterized by vigor, dedication, and absorption (Schaufeli and Bakker, 2004). Engaged workers put more effort in their jobs, and consequently perceive higher employer obligations than do unengaged workers (Matthijs Bal et al., 2011). Vigor refers to high levels of energy and resilience in work. Dedication is characterized by strong involvement in one’s work as well as a sense of significance and enthusiasm. Absorption is a state of being fully concentrated and happily engrossed in one’s work. Hence, engaged employees are usually equipped with high levels of energy and are enthusiastically involved in their work (Yongxing et al., 2017).

Because emotional job demands, when in abundance, can become stressors and cause emotional exhaustion as proposed by the JD-R model, we can reasonably expect that employees’ vigor, dedication, and disposition are specific casualties in such situations. As such, in the link between emotional job demands and absenteeism likelihood, work engagement may play a significant contributing role. Accordingly, we propose the following hypothesis.

Hypothesis 2: Work engagement mediates the link between emotional job demands and absenteeism likelihood.

### The Moderating Role of Regulatory Institutional Environments

The regulatory institutional environment refers to a system of legal rewards and sanctions that ensure compliance behavior (Robert, 1974; Meyer and Rowan, 1977). Institutional theory has been acknowledged as a powerful perspective to examine various phenomena, and it is important in that institutions have the ability to either legitimize or constrain the actions of both individuals and firms (Kreiser et al., 2002). Institutions differ from context to context; in other words, there is no general set of accepted institutions that is applicable to all instances of a particular behavior. The cost-benefit model tells us that individuals are rational beings who carefully calculate the pros and cons of personal decisions (Pearce, 1984; Margalit, 2011). The model assumes that rational decision-makers act in order to maximize their expected utility such that they will consequently cease an activity when they estimate that its costs exceed its benefits. Following the basic ideas of this model, we argue that even though, according to the findings of previous studies, employee work engagement negatively affects absenteeism (Schaufeli and Rhenen, 2009; Soane et al., 2013), employees take into consideration regulatory institutional environments before engaging in absenteeism behavior. Regulatory institutions impose or prescribe standards of acceptable behavior and set the legal penalties for non-observance of those standards. We more specifically argue that the regulatory institutional environment plays a role in moderating the link between work engagement and employee-absenteeism decisions. Accordingly, we propose the following hypothesis.

Hypothesis 3: Regulatory institutional environments moderate the negative link between work engagement and absenteeism likelihood.

**FIGURE 1:**
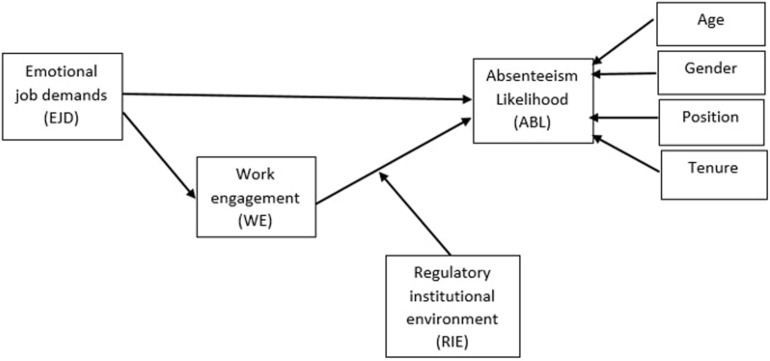
Proposed study framework.

Figure 1 presents the current study’s research model, which rests on the sum of the hypotheses proposed herein.

## Materials and Methods

### Participants

The population of the current study is healthcare workers in the low-income country of Malawi. We chose Malawi because it was amongst the poorest countries in the world according to World Bank data in 2019^1^. We collected the data by using a convenient non-probability-sampling method in which people are sampled simply because they are “convenient” sources of data for researchers (Lancaster, 2005). We chose healthcare workers as the target population for this study because they have the highest rate of absenteeism in Malawi’s workforce (Manafa et al., 2009). A total of 496 healthcare workers replied to this study questionnaire, thus representing a response rate of 82.83%. After screening the data, we removed a total of 9 responses as they were incomplete, thus reducing the responses for processing to 487. Of these respondents, 211 were females and 276 were males, representing 43.33 and 55.65%, respectively. In terms of age, 95 respondents were under 25 years old, 28 respondents were between 25 and 29, 41 respondents were between 30 and 34, 137 respondents were between 35 and 39, 129 respondents were between 40 and 44, and 47 respondents were 45 years old or older. These age-related categories account for 19.5, 5.7, 8.4, 30.2, 26.5, and 9.7% of the total pool respectively.

### Procedure

First, we communicated the research objectives to all the participants and guaranteed both their anonymity and our compliance with the ethical standards governing confidentiality in data processing. In 2018, the questionnaires used for data collection were implemented on a Web platform, which enabled the respondents to fill out the questionnaires online. About 600 questionnaires were distributed through the healthcare workers’ staff emails, and the questionnaires were all answered online. We included a series of control questions in the questionnaire to detect random and incongruent answers. We discarded incomplete and incongruent questionnaires from the study sample.

### Instruments

#### Absenteeism Likelihood

In this study, rather than use employees’ organization records, we used a self-reported proxy to measure for absenteeism likelihood because organizations in such low-income countries as Malawi (our research setting) are unlikely to have accurate absenteeism records. Prior studies have stated that the unavailability of an organization’s absenteeism records is a common problem for scholars seeking to measure employee absenteeism, so we have solved the problem by using a self-reported proxy (Johns, 1994, 2003). Johns and Miraglia (2015) concluded that self-reports of absenteeism offer adequate test-retest reliability and that they exhibit reasonably good rank order. Because the focus of our measurements in the current study is on the likelihood of voluntary absenteeism as opposed to involuntary absenteeism, we employ only 5 measurement items out of the 13 items adopted from Paget et al. (1998). This proxy of absenteeism likelihood was originally developed by Nicholson and Payne (1987). Respondents identify the likelihood that each of five common factors might result in an absence from work. An aggregate of their responses serves as an estimate for their absenteeism likelihood. One question corresponds to each of the five factors: (1) “How likely is it that you would be voluntarily absent from work because you are feeling depressed?” (ABL1), (2) “How likely is it that you would be voluntarily absent from work because you had a fallout with your workmates or supervisor?” (ABL2), (3) “How likely is it that you would be voluntarily absent from work because the schedule of a personal activity conflicts with your work schedule?” (ABL3), (4) “How likely is it that you would be voluntarily absent from work because you did not wake up on time to go to work?” (ABL4), and (5) “How likely is it that you would be voluntarily absent from work because you are experiencing minor domestic problems?” (ABL5). We used a 5-point Likert scale ranging from “1 = Not at all” to “5 = Highly likely.”

#### Emotional Job Demands

We measured emotional job demands by using six items adopted from Xanthopoulou et al. (2013). Emotional job demands are also a component of the JD-R model. Questionnaire items include (1) “Is your work emotionally demanding?” (EJD1), (2) “Do you face emotionally challenging situations at your work?” (EJD2), (3) “In your work, are you confronted with things that personally touch you?” (EJD3), (4) “In your work, do you deal with clients who incessantly complain, although you always do everything to help them?” (EJD4), (5) “Do you have to deal with clients who do not treat you with the appropriate respect and politeness?” (EJD5), and (6) “In your work, do you have sentimental experiences?” (EJD6). We used a five-point Likert scale ranging from “1 = Never” to “5 = Always.”

#### Work Engagement

We measured work engagement by using nine items adopted from Schaufeli and Bakker (2004). There questionnaire items are: (1) “At my work, I am bursting with energy” (WE1), (2) “At my job, I feel strong and vigorous” (WE2), (3) “When I get up in the morning, I feel like going to work” (WE3), (4) “I am enthusiastic about my job” (WE4), (5) “At my job, I feel strong and vigorous (WE5),” (6) “I feel happy when I am working intensely” (WE6), (7) “My job inspires me” (WE7), (8) “I am immersed in my work” (WE8), and (9) “I get carried away when I am working” (WE9). We used a five-point Likert scale ranging from “1 = Never” to “5 = Always.”

#### Regulatory Institutional Environment

We measured regulatory institutional environment by using three items that we had specifically developed for this study on the basis of previous studies: Bamberger and Michal (2007); Busenitz et al. (2000), Kostova (1997), and Parboteeah et al. (2008). Because no general set of accepted institutions (regulatory, normative, and cognitive) is applicable to all contexts of a particular behavior, we employed some items that better fit our current research context (Merton, 1968; Robert, 1974; Meyer and Rowan, 1977). We concurred with Kostova (1997) and Roxas and Coetzer (2012), who argued that each study should develop its own items to measure a particular construct in an institutional environment so that in this way the items can be domain specific. For our current study, we conducted interviews with fifteen health experts in Malawi to help confirm if the items developed represented the existing regulatory institutional environment related to healthcare workers’ absenteeism in a hospital setting. The interviews conducted confirmed that those three items of the regulatory institutional environment were related to healthcare workers’ absenteeism and represented a system of sanctions and rewards aimed at ensuring healthcare workers’ compliance with work attendance behavior. We used questionnaire items: (1) “Being voluntarily absent from my work will lead to a reduction in my wages” (RIE1), (2) “Being voluntarily absent from my work will lead to disciplinary measures” (RIE2), and (3) “Being voluntarily absent from my work will lead to reductions in my promotion opportunities” (RIE3). We used a 5-point Likert scale ranging from “1 = Strongly disagree” to “5 = Strongly agree.”

#### Control Variables

Drawing on previous research, we controlled for respondents’ age, gender (1 = Male, 2 = Female), position, and tenure. We extracted the data from the biographical information provided by respondents on the study questionnaire. We measured age on the basis of a single question (“How old are you?”) to which the respondents replied by simply filling in the appropriate age. We measured position by having respondents select one of three options: (1) junior, (2) senior, and (3) supervisor. We measured tenure by having respondents select one of six options: (1) less than 1 year, (2) 1 to less than 2 years, (3) 2 to 3 less than years, (4) 3 to less than 5 years, (5) 5 to less than 10 years, or (6) more than 10 years.

### Pilot Test

In order to test for the reliability of the study’s construct prior to conducting the main survey, we conducted a pilot study. During the pilot study, a sample of 125 healthcare workers responded to the test questionnaire that we had sent them via email. Their ages ranged from 24 to 48. The pilot-study results indicated that all the study constructs were reliable for use in the study: emotional job demands (Cronbach α = 0.778), work engagement (Cronbach α = 0.845), regulatory institutional environment (Cronbach α = 0.774), and absenteeism likelihood (Cronbach α = 0.865). From the pilot test results, it can be seen that all the study constructs had Cronbach’s Alphas greater than 0.7, indicating sufficient reliability of the study constructs (Patten, 2000; Creswell, 2002).

### Data-Analysis Strategy

We used structural equation modeling (SEM) and the analytical tool of AMOS 21 to test our research model in this study. To further test the significance of the mediating effect, we employed bootstrap analysis. We empirically tested the moderation effect by using moderated regression and the analytical tool of SPSS 21. Before undertaking further data analyses, we processed each study variable by performing data cleaning and imputation (via the mean value of each item). In this way, we infused our data with sufficient robustness.

### Multicollinearity Assessment

We initially conducted a multicollinearity assessment in order to rule out the possibility of multicollinearity issues. To this end, we examined variance inflation factors (VIFs). Hair et al. (1995) recommended that multicollinearity is a concern if a VIF value is higher than 10, as such a value inflates the variance of regression parameters and can thus lead to the wrong identification of relevant predictors in a statistical model. The results of our multicollinearity analysis reveal that all study constructs had VIF values less than 10. In other words, our study suffered from no multicollinearity issues. In fact, the highest VIF value observed was 1.117 in this study.

### Common Method Bias

The current study design is cross-sectional in nature and may suffer from mono-methodological bias (Podsakoff et al., 2003, 2012). We thus conducted Harman’s single factor test in order to rule out common method bias (Harman, 1976). The test, in general, explains that common method bias is present when only one factor emerges or when one factor accounts for more than 50% of the variance associated with all items loaded simultaneously in a factor analysis (Harman, 1976). The outcome of the current study’s factor analysis shows that a single factor explained 34.923% of the total variance. The result indicates that no single factor accounted for more than 50% of the variance of all the simultaneously loaded items. This leads to our conclusion that there was no common method bias in our study’s data set.

## Results

Following the suggestions offered by Anderson and Gerbing (1988) on structural equation modeling (SEM), we used a two-stage approach in order to test for our model in AMOS 21. First, we conducted a confirmatory factor analysis to assess the quality of the measurement model. Second, we used a structural model to test our hypotheses. We then, using the bootstrap method, conducted a further analysis pertaining to the significance of the mediating effect. We tested for the moderating effect by using moderated regression analysis in SPSS.

### Measurement Model

There are four latent factor (emotional job demands, work engagement, regulatory institutional environment, and absenteeism likelihood) and 18 observed variables. In the first step, we conducted an exploratory factor analysis of each of the constructs individually to test for uni-dimensionality as shown in Table 1 (Anderson and Gerbing, 1988). We discarded any item that had a factor loading less than 0.6 (Bagozzi and Yi, 1988). The eliminated items are 0.518 for EJD6, 0.381 for WE1, 0.559 for WE2, 0.555 for WE8, and 0.307 for WE9. As Table 1 shows, the composite reliability values ranging from 0.571 to 0.741 were greater than (or approaching) the suggested cut-off of 0.6 (Bagozzi and Yi, 1988). The average variance extracted (AVE) values fell into a range between 0.867 and 0.934. All of these values are greater than the threshold of 0.5. Moreover, all factor loadings were greater than the threshold value of 0.6. Overall the results indicate satisfactory reliability regarding the latent variables. We checked the discriminant validity of the latent variables. In Table 2, the values of the square roots of the AVE were all greater than the correlated coefficient values between any two constructs illustrating that our data achieved satisfactory discriminant validity (Espinoza, 1999).

**TABLE 1:** Exploratory factor analysis.

Constructs and measures	Average	SD	Standardized loadings	Construct reliability (CR)	Average variance extracted (AVE)
**Emotional job demands**					
EJD1	3.72	1.325	0.804	0.639	0.898
EJD2	3.69	1.383	0.864		
EJD3	3.74	1.357	0.885		
EJD4	3.99	1.192	0.723		
EJD5	4.04	1.2	0.703		
**Work engagement**					
WE3	2.51	1.079	0.675	0.571	0.867
WE4	2.28	1.023	0.745		
WE5	2.19	1.098	0.877		
WE6	2.07	1.042	0.848		
WE7	2.33	1.031	0.595		
**Absenteeism**					
AB1	3.71	1.36	0.817	0.741	0.934
AB2	3.75	1.553	0.951		
AB3	3.72	1.55	0.962		
AB4	3.19	1.321	0.718		
AB5	3.53	1.294	0.829		
**Regulatory institutions**					
RIE1	3.59	1.596	0.735	0.669	0.858
RIE2	4.13	1.191	0.893		
RIE3	4.15	1.196	0.818		

**TABLE 2:** Descriptive statistics, correlations, and reliability.

Constructs	1	2	3	4
1. Emotional job demands	***0.948***			
2. Work engagement	−0.153**	***0.931***		
3. Absenteeism	0.560**	−0.262**	***0.966***	
4. Regulatory institutions	−0.170**	0.137**	−0.341**	***0.926***

*The bold and italicized cells display the values of the squared averaged variance extracted, while others show the values of the correlated coefficients between our constructs.*

We then deployed CFA by comparing four models to distinguish study variables including emotional job demands, work engagement, regulatory institutional environment, and absenteeism likelihood. We compared one-factor model (all observed variables loaded on a single factor), two-factor model (emotional job demands and work engagement as one factor and regulatory institutional environment and absenteeism likelihood as the other), three-factor model (emotional job demands and work engagement as one factor and regulatory institutional environment and absenteeism likelihood as separate factors), and four-factor model (emotional job demands, work engagement, regulatory institutional environment, and absenteeism likelihood as separate factors). CFA results revealed that the four-factor model demonstrated the better fit as shown in Table 3. This measurement model showed a better fit: χ^2^ (219, *N* = 487) = 785.06; RMSEA = 0.073, CFI = 0.945, IFI = 0.940, TLI = 0.925. In Table 3, a comparison of the Δχ^2^ also reveals that the four-factor model is superior to all other models.

**TABLE 3:** Fit indices for the measurement models (*N* = 487).

	χ^2^	df	χ^2^/df	Δχ^2^ (df)	*p*	RMSEA	CFI	IFI	TLI
One factor	3475.1	275	12.637			0.155	0.692	0.694	0.625
Two factor	2349.32	269	8.73			0.126	0.797	0.698	0.640
Three factor	1173.15	235	4.99			0.091	0.862	0.863	0.817
Four factor	785.06	219	3.58			0.073	0.945	0.940	0.925
One factor vs. 2				1125.78 (6)	0.05				
One factor vs. 3				2301.95 (40)	0.05				
One factor vs. 4				2690.04 (54)	0.05				
Two factor vs. 3				1176.17 (34)	0.05				
Two factor vs. 4				1564.26 (50)	0.05				
Three factor vs. 4				388.09 (16)	0.05				

*RMSEA, root mean square error of approximation; CFI, comparative fit index; IFI, incremental fit index; and TLI, Tucker Lewis Index. A comparison of the change in χ^2^ shows that they are all significantly different meaning that the four-factor model is superior to all others.*

### Structural Model With Work Engagement as a Mediator

**FIGURE 2:**
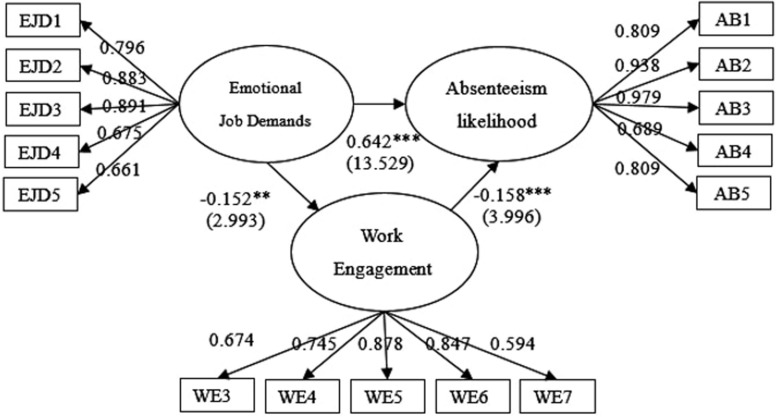
The structural model for emotional job demands, work engagement, and absenteeism likelihood.

We created our measurement model after conducting validity and reliability tests in a CFA. Further, we developed a structural model to test the proposed hypotheses in this study. We employed AMOS 21 to conduct a path analysis. Figure 2 shows the results from the analysis: they present the path coefficient from the independent constructs to their corresponding constructs as stated in the research hypotheses. Overall, the structural model shows acceptable goodness of fit with χ^2^/df = 3.001, RMSEA = 0.078, GFI = 0.937, CFI = 0.972, NFI = 0.959, PCFI = 0.750, IFI = 965 and PNFI = 0.739. Furthermore, the results of SEM show that emotional job demands had significant and positive effects on absenteeism likelihood with a β coefficient of 0.642; these results thus support H1. Furthermore, the influence of emotional job demands on work engagement was significant and negative (β coefficient = −0.152, *t*-value = 2.993). The influence of work engagement on absenteeism likelihood was significant and negative (β coefficient = −0.158, *t*-value = 3.996). The results of the comparison between the β of direct and indirect paths of work engagement as a mediator in the link between emotional job demands and absenteeism likelihood show that the indirect effects of 0.024 (i.e., −0.158 ^∗^ −0.152) are less than the direct effects of 0.642. This result indicates that work engagement is a significant mediator in the relationship; the overall results thus support H2.

We conducted a bootstrap analysis to further test the extent to which work engagement had a mediating effect on the link between emotional job demands and absenteeism likelihood. This post-SEM analysis has been adopted by many scholars, such as Gong et al. (2020) and Nauman et al. (2018) with the aim of strengthening the initial mediating-effects results. Bootstrap samples (5,000) were generated from the original sample set (*N* = 487) through random sampling. The absence of zero in the 99% confidence interval for the estimates indicates that the mediation effect was significant (Cheung and Lau, 2008). Table 4 demonstrates the mediating effects that work engagement have in the link between emotional job demands and absenteeism likelihood. Table 4 also shows the results of the mediation analysis. As indicated in Table 4, work engagement exerted significant indirect effects on the link between emotional job demands and absenteeism likelihood offering further credence to H2.

**TABLE 4:** Bootstrap results for total, direct and indirect effect.

	Path	Estimated Effect	LL 99% CI	UL 99% CL
Total Effect	EJD → ABL	0.6470	0.5346	0.7593
Direct Effect	EJD → ABL	0.6150	0.5038	0.7262
Indirect Effect	EJD → WE→ ABL	0.0320	0.0068	0.0692

*Empirical 99% confidence interval does not overlap with zero. CI, confidence interval; EJD, emotional job demands; WE, work engagement; ABL, absenteeism likelihood.*

### The Moderating Effect of Regulatory Institutions

**FIGURE 3:**
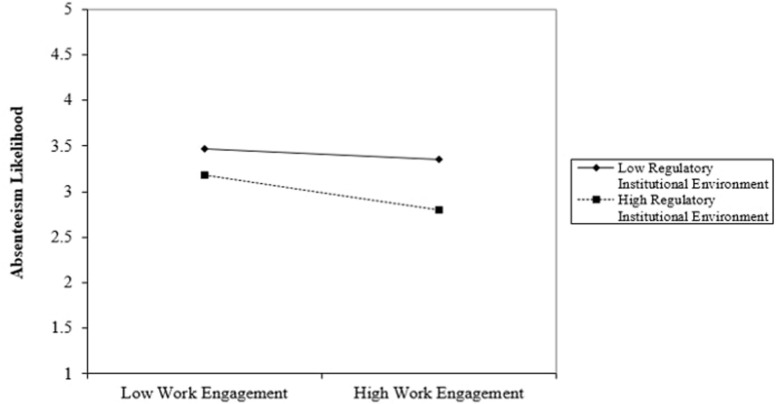
Moderating effect of regulatory institutional environment in the relationship between work engagement, and absenteeism likelihood.

Hypothesis 3 predicts that regulatory institutional environment moderates the negative link between work engagement and absenteeism likelihood. We conducted a moderated regression analysis to analyze this moderating effect. Table 5 presents the results. The results show that the interaction term has a statistically significant coefficient (β = −0.081, *p* < 0.05), implying that regulatory institutional environment has a moderating effect on the negative link between work engagement and absenteeism likelihood. Figure 3 presents the graphical plot of the moderating effect of regulatory institutions on the link between work engagement and absenteeism likelihood. In the plot, the moderating effect is significantly higher in the high regulatory institutional environment than in the low one.

**TABLE 5:** Moderated regression analysis results.

Variables	β
**Controls**	
Gender	–0.035
Age	–0.099
Position	–0.037
Tenure	0.049
**Predictors**	
Emotional job demands	0.465***
Work engagement (WE)	−0.150***
**Moderator**	
Regulatory Institutional Environment (RIE)	−0.230***
**Interaction Effect**	
WE × RIE	−0.081**
*R*^2^ (Adjusted *R*^2^)	0.421 (0.411)

** *P* < 0.05; *** *P* < 0.01. Regression results above are for the complete model with all the variables entered at the same time.

## Discussion

The results of our current study not only demonstrate the positive link between the emotional job demands and the absenteeism likelihood of healthcare workers but also highlight important possible work-engagement interventions that management can adopt to lessen the effects of emotional job demands on employee-absenteeism likelihood. Managers should consider not only stiffening the regulatory institutions pertaining to absenteeism but also increasing employees’ awareness of the regulatory institutions as these steps will very likely lessen the negative effect of low work engagement on employee-absenteeism likelihood.

This study also makes a number of contributions to the theoretical side of this issue. First, this study has enriched the JD-R model as it concerns employee absenteeism in the context of low-income countries. Our primary hypothesis suggested that emotional job demands positively relate to employee-absenteeism likelihood, and our findings indeed demonstrate the existence of this link in the context of low-income countries. Second, this article has incorporated and clearly demonstrates the role of the regulatory institutional environment in the effect of emotional job demands on employee-absenteeism likelihood. To demonstrate this role, we tested specifically the moderating role played by the regulatory institutional environment in the link between work engagement (an outcome of emotional job demands) and employee-absenteeism likelihood. In so doing, we have brought into clearer focus the pertinent roles played by context-specific regulatory institutional environments. Third, because past research has found that low-income countries’ regulatory institutional environments are weak, it is worth noting that – according to our current study – these environments still matter as they moderate the negative effect of work engagement on absenteeism likelihood. Fourth, by conducting a survey in a low-income country, we have extended the geographical reach of research on employee absenteeism. Fifth, this study has demonstrated the applicability of the JD-R model to low-income countries and to regulatory institutional environments. Because the economies in low-income countries are markedly different from those in developed and emerging economies, this study sheds considerable light on how emotional job demands, work engagement, and regulatory institutional environment all interact to predict employee absenteeism.

### Limitations and Future Research Directions

As with all studies, the current one has its limitations. The first one concerns this study’s absenteeism-likelihood measurement item, which requires respondents to self-assess the likelihood of their being absent across five common reasons. In reality, there are more than just five reasons underlying employees’ decision to be absent from work. The results of this study should be carefully interpreted in light of this fact. Further studies can test the current study’s findings by using a different measure of employees’ absenteeism. A second limitation is time, as we had to collect the results during a disappointingly short period (Collins et al., 2015). Another limitation comes from linguistic problems that arose: although English is the official language of Malawi, we could not guarantee that every respondent had sufficiently high English-comprehension skills when interpreting the questionnaire’s wording. Lastly, in this study, we collected the data of all variables from the same group of respondents in one-time period via a cross-sectional survey. We chose this approach owing to the difficulty of collecting data from healthcare workers in two time periods. This data-collection approach may suffer from common method variance (Podsakoff et al., 2003). However, it bears repeating that we conducted a Harman’s single factor test to rule out the problem. Additional studies would do well to develop a normative approach relative to regulatory institutional environment in documenting the problem of absenteeism in the low-income country context. Such studies will offer additional clarity to our understanding of how employee absenteeism manifests itself in low-income countries.

## Conclusion

This study has offered additional insights into how emotional job demands relate positively to employee-absenteeism likelihood in the context of low-income countries. In general, the greater the emotional demands of a job are, the higher the absenteeism likelihood of the employees will be. Work engagement mediates the effect of emotional job demands on employee-absenteeism likelihood, and this mediation highlights the important role of workplace attitudes in explaining the effects of emotional job demands on employee absenteeism. But most important to note is the moderating role of the regulatory institutional environment in the negative link between work engagement (an outcome of emotional job demands) and absenteeism likelihood. This finding demonstrates how regulatory institutional environments moderate the link between emotional job demands and employee-absenteeism likelihood when considered through the work-engagement pathway.

## Data Availability Statement

The datasets generated for this study are available on request to the corresponding author.

## Ethics Statement

The studies involving human participants were reviewed and approved by Research Committee at the National Dong Hwa University. The patients/participants provided their written informed consent to participate in this study.

## Author Contributions

BM carried out the data collection and analysis, and wrote the manuscript. Y-YC helped with the designing of the study framework and edited the manuscript. W-CC helped with analysis of the data set and interpretation of the results.

## Conflict of Interest

The authors declare that the research was conducted in the absence of any commercial or financial relationships that could be construed as a potential conflict of interest.

## Footnotes

1 https://www.worldbank.org/en/country/malawi/overview
